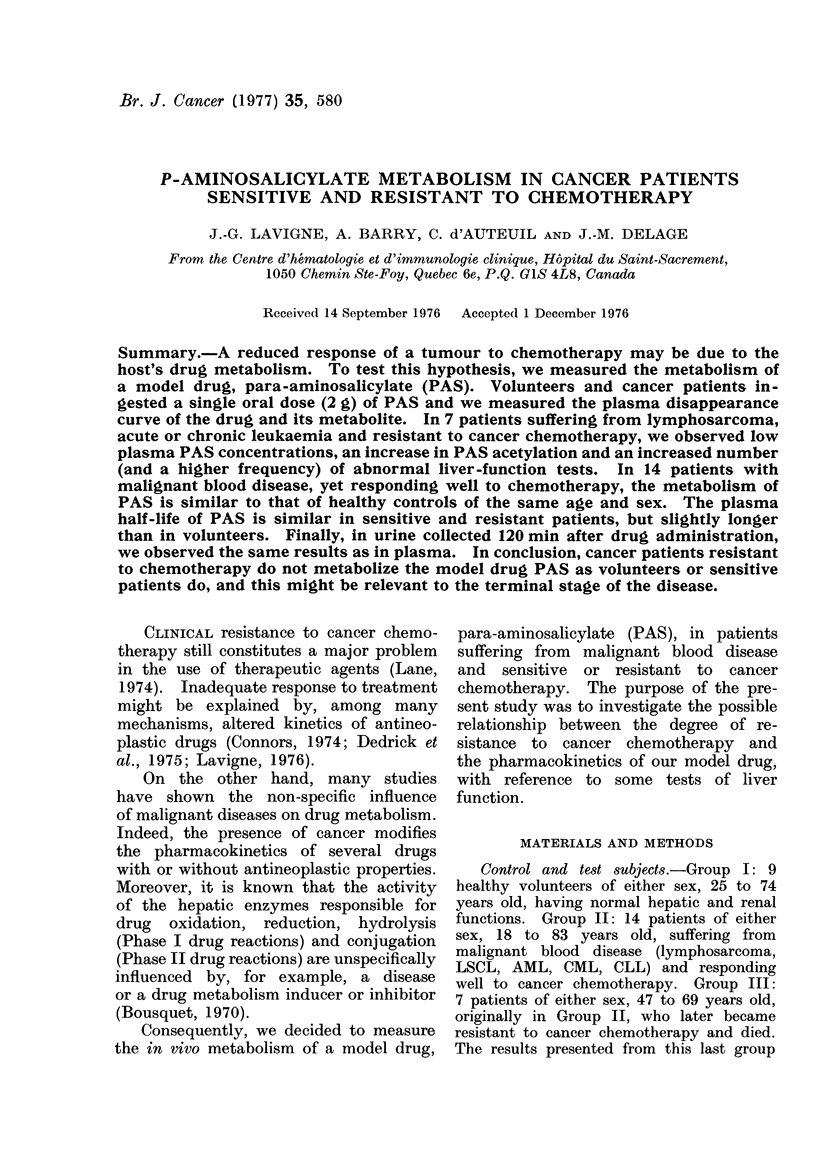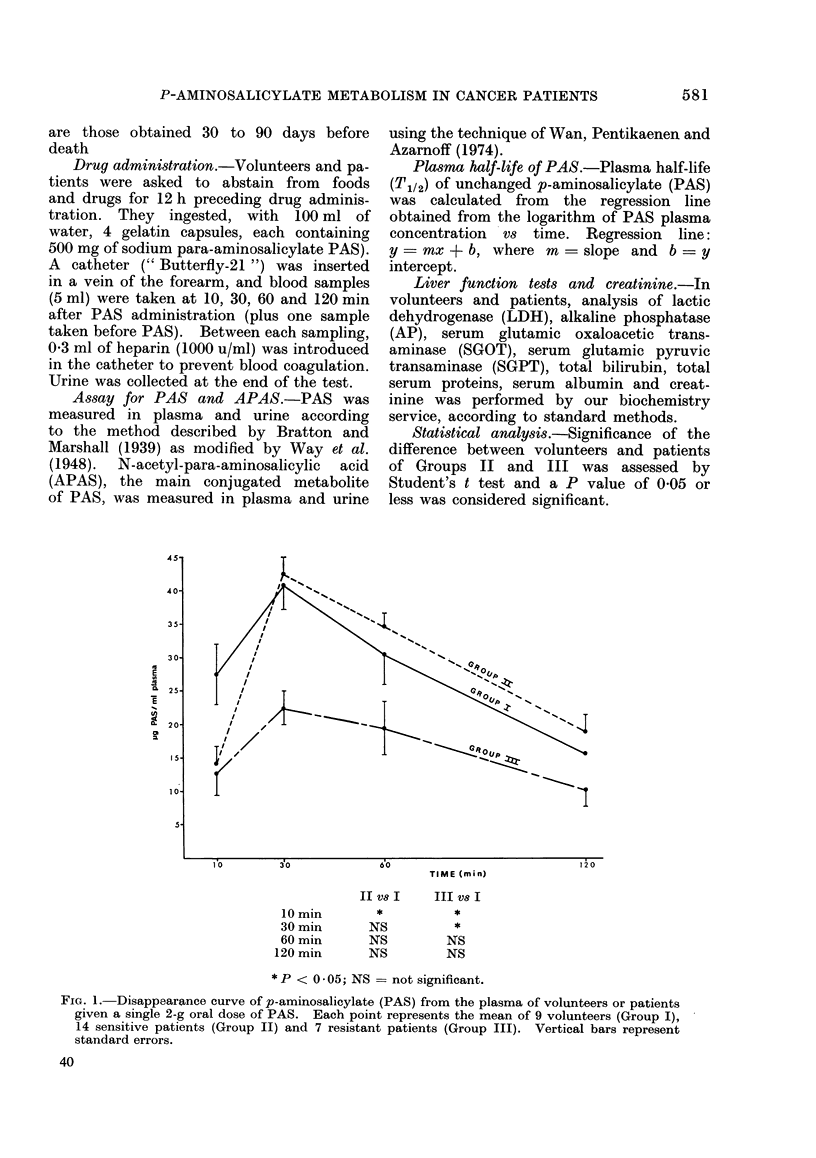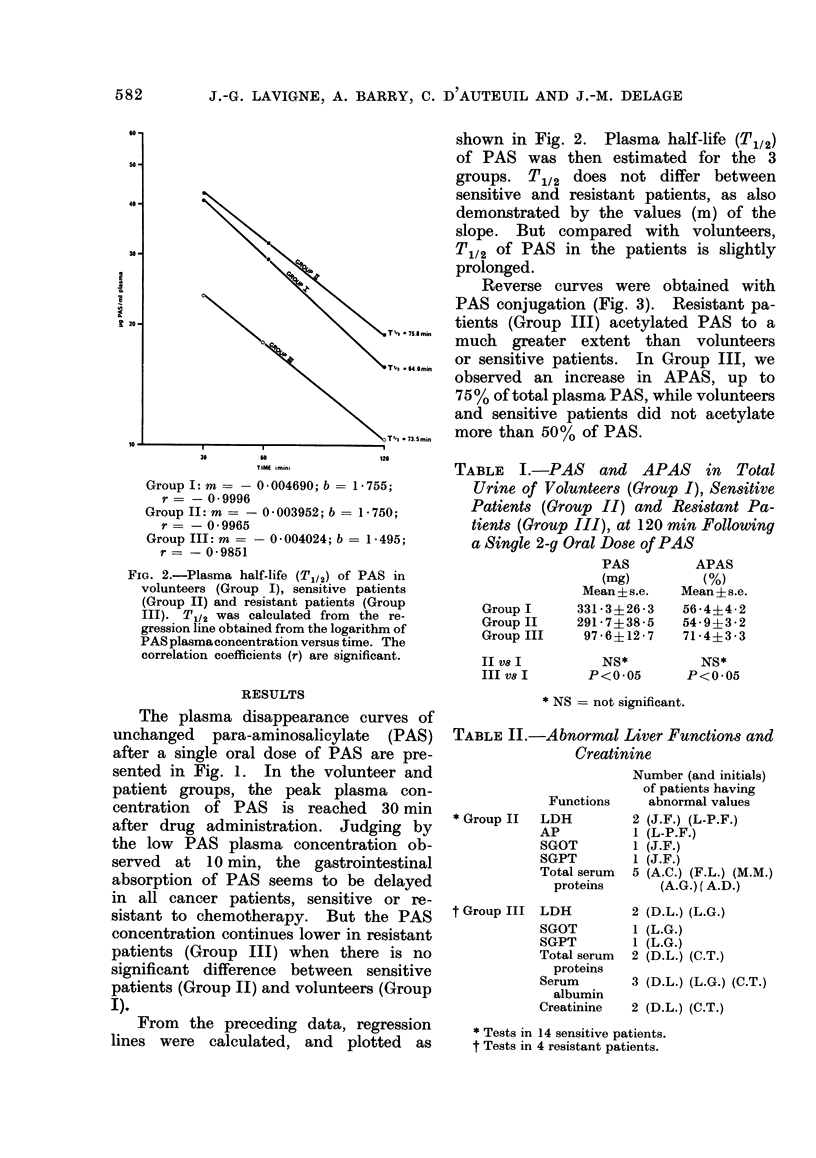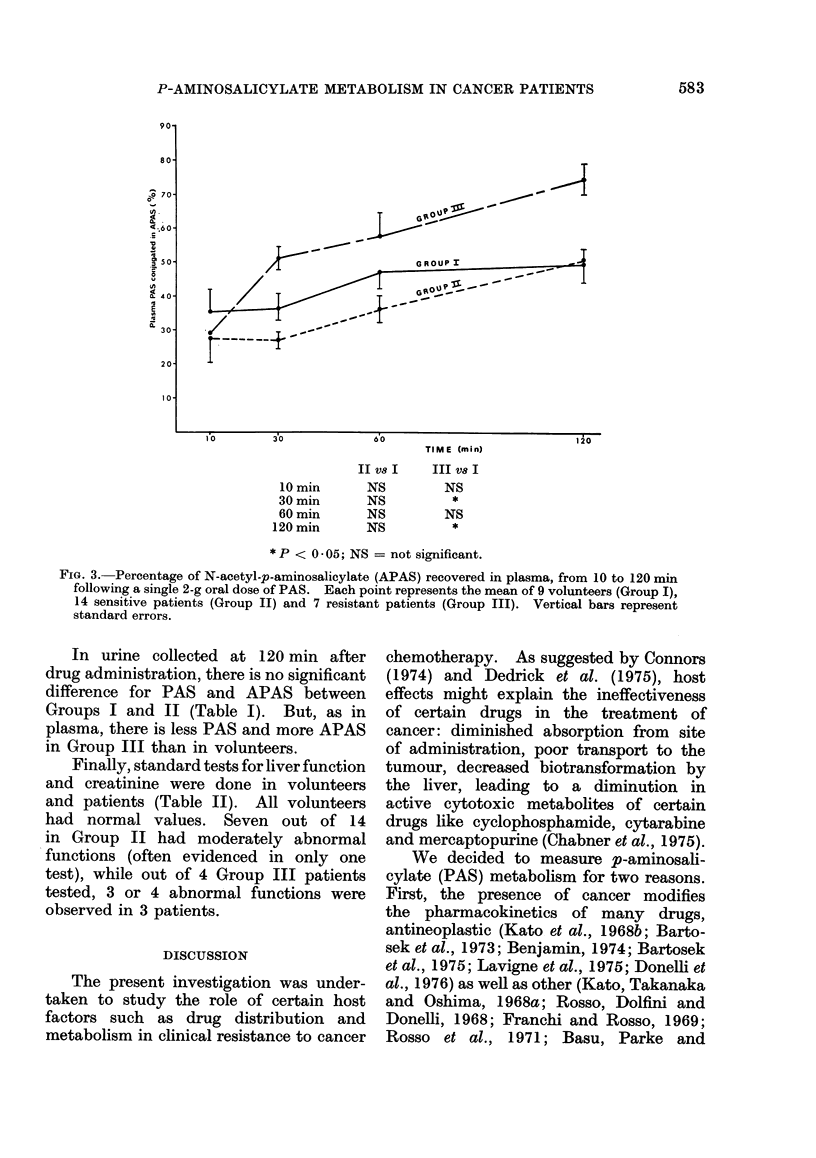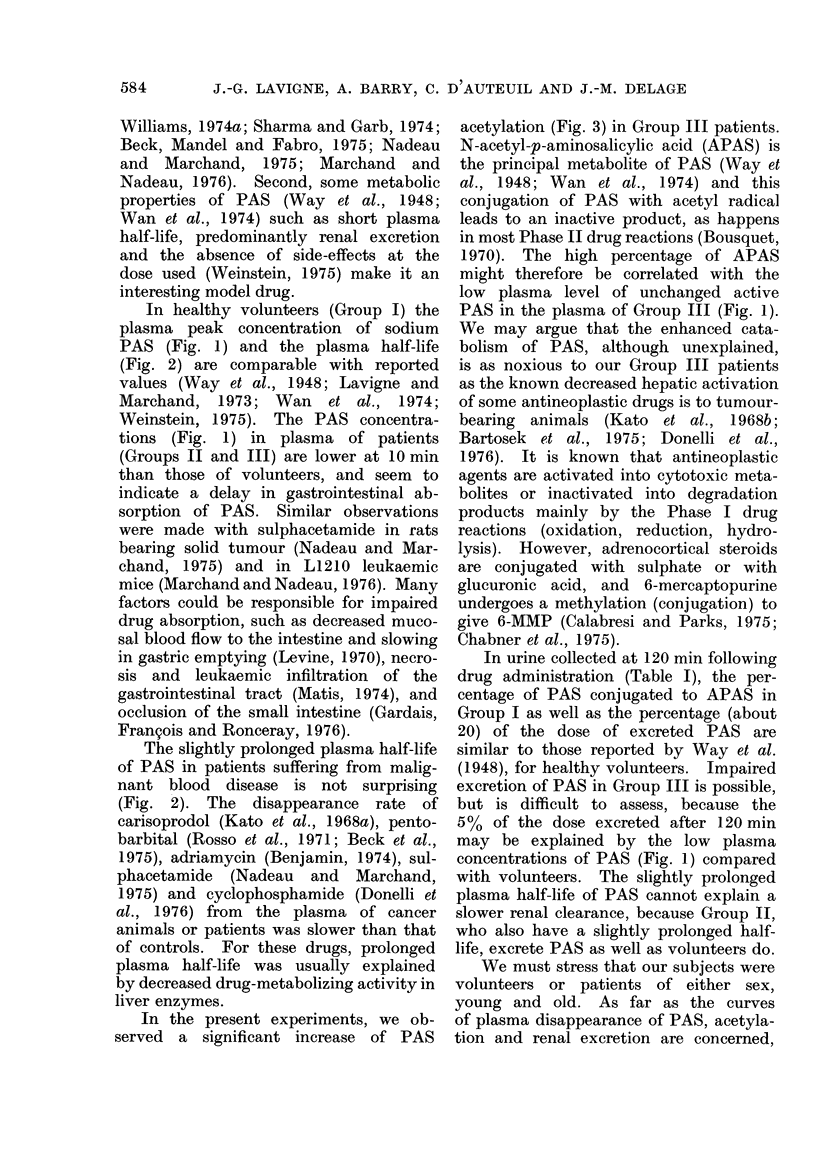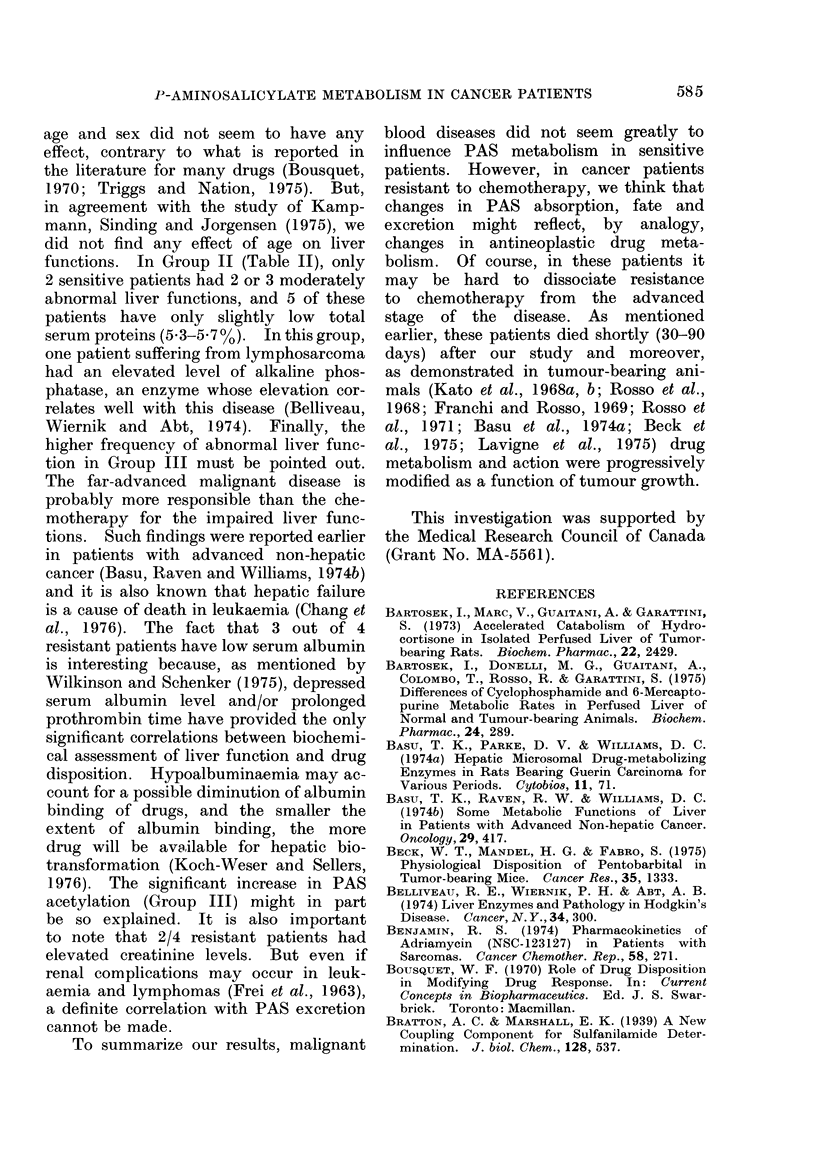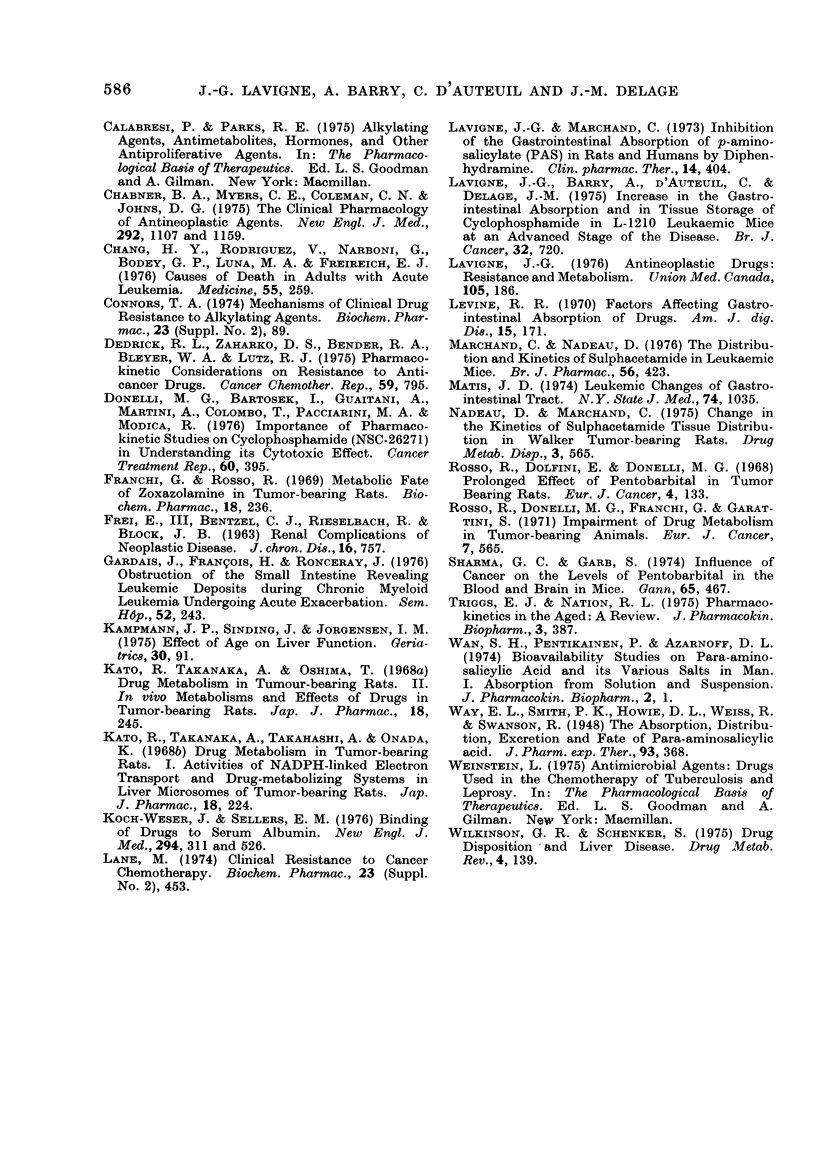# P-aminosalicylate metabolism in cancer patients sensitive and resistant to chemotherapy.

**DOI:** 10.1038/bjc.1977.91

**Published:** 1977-05

**Authors:** J. G. Lavigne, A. Barry, C. d'Auteuil, J. M. Delage

## Abstract

A reduced response of a tumour to chemotherapy may be due to the host's drug metabolism. To test this hypothesis, we measured the metabolism of a model drug, para-aminosalicylate (PAS). Volunteers and cancer patients ingested a single oral dose (2 g) of PAS and we measured the plasma disappearance curve of the drug and its metabolite. In 7 patients suffering from lymphosarcoma, acute or chronic leukaemia and resistant to cancer chemotherapy, we observed low plasma PAS concentrations, an increase in PAS acetylation and an increased number (and a higher frequency) of abnormal liver-function tests. In 14 patients with malignant blood disease, yet responding well to chemotherapy, the metabolism of PAS is similar to that of healthy controls of the same age and sex. The plasma half-life of PAS is similar in sensitive and resistant patients, but slightly longer than in volunteers. Finally, in urine collected 120 min after drug administration, we observed the same results as in plasma. In conclusion, cancer patients resistant to chemotherapy do not metabolize the model drug PAS as volunteers or sensitive patients do, and this might be relevant to the terminal stage of the disease.


*Br. J. Cancer* (1977) **35**, 580

### *P*-AMINOSALICYLATE METABOLISM IN CANCER PATIENTS SENSITIVE AND RESISTANT TO CHEMOTHERAPY

J.-G. LAVIGNE, A. BARRY, C. d'AUTEUIL AND J.-M. DELAGE

*From the Centre d'hématologie et d'immunologie clinique, Hôpital du Saint-Sacrement, 1050 Chemin Ste-Foy, Quebec 6e, P.Q. G1S 4L8, Canada*

Received 14 September 1976  Accepted 1 December 1976

**Summary.—A reduced response of a tumour to chemotherapy may be due to the host's drug metabolism. To test this hypothesis, we measured the metabolism of a model drug, para-aminosalicylate (PAS). Volunteers and cancer patients ingested a single oral dose (2 g) of PAS and we measured the plasma disappearance curve of the drug and its metabolite. In 7 patients suffering from lymphosarcoma, acute or chronic leukaemia and resistant to cancer chemotherapy, we observed low plasma PAS concentrations, an increase in PAS acetylation and an increased number (and a higher frequency) of abnormal liver-function tests. In 14 patients with malignant blood disease, yet responding well to chemotherapy, the metabolism of PAS is similar to that of healthy controls of the same age and sex. The plasma half-life of PAS is similar in sensitive and resistant patients, but slightly longer than in volunteers. Finally, in urine collected 120 min after drug administration, we observed the same results as in plasma. In conclusion, cancer patients resistant to chemotherapy do not metabolize the model drug PAS as volunteers or sensitive patients do, and this might be relevant to the terminal stage of the disease.**

CLINICAL resistance to cancer chemotherapy still constitutes a major problem in the use of therapeutic agents (Lane, 1974). Inadequate response to treatment might be explained by, among many mechanisms, altered kinetics of antineoplastic drugs (Connors, 1974; Dedrick *et al.*, 1975; Lavigne, 1976).

On the other hand, many studies have shown the non-specific influence of malignant diseases on drug metabolism. Indeed, the presence of cancer modifies the pharmacokinetics of several drugs with or without antineoplastic properties. Moreover, it is known that the activity of the hepatic enzymes responsible for drug oxidation, reduction, hydrolysis (Phase I drug reactions) and conjugation (Phase II drug reactions) are unspecifically influenced by, for example, a disease or a drug metabolism inducer or inhibitor (Bousquet, 1970).

Consequently, we decided to measure the *in vivo* metabolism of a model drug, para-aminosalicylate (PAS), in patients suffering from malignant blood disease and sensitive or resistant to cancer chemotherapy. The purpose of the present study was to investigate the possible relationship between the degree of resistance to cancer chemotherapy and the pharmacokinetics of our model drug, with reference to some tests of liver function.

### MATERIALS AND METHODS

*Control and test subjects.*—Group I: 9 healthy volunteers of either sex, 25 to 74 years old, having normal hepatic and renal functions. Group II: 14 patients of either sex, 18 to 83 years old, suffering from malignant blood disease (lymphosarcoma, LSCL, AML, CML, CLL) and responding well to cancer chemotherapy. Group III: 7 patients of either sex, 47 to 69 years old, originally in Group II, who later became resistant to cancer chemotherapy and died. The results presented from this last group are those obtained 30 to 90 days before death

*Drug administration.*—Volunteers and patients were asked to abstain from foods and drugs for 12 h preceding drug administration. They ingested, with 100 ml of water, 4 gelatin capsules, each containing 500 mg of sodium para-aminosalicylate PAS). A catheter (" Butterfly-21 ") was inserted in a vein of the forearm, and blood samples (5 ml) were taken at 10, 30, 60 and 120 min after PAS administration (plus one sample taken before PAS). Between each sampling, 0·3 ml of heparin (1000 u/ml) was introduced in the catheter to prevent blood coagulation. Urine was collected at the end of the test.

*Assay for PAS and APAS.*—PAS was measured in plasma and urine according to the method described by Bratton and Marshall (1939) as modified by Way *et al.* (1948). N-acetyl-para-aminosalicylic acid (APAS), the main conjugated metabolite of PAS, was measured in plasma and urine using the technique of Wan, Pentikaenen and Azarnoff (1974).

*Plasma half-life of PAS.*—Plasma half-life ($T_{1/2}$) of unchanged *p*-aminosalicylate (PAS) was calculated from the regression line obtained from the logarithm of PAS plasma concentration *vs* time. Regression line: $y = mx + b$, where $m$ = slope and $b = y$ intercept.

*Liver function tests and creatinine.*—In volunteers and patients, analysis of lactic dehydrogenase (LDH), alkaline phosphatase (AP), serum glutamic oxaloacetic transaminase (SGOT), serum glutamic pyruvic transaminase (SGPT), total bilirubin, total serum proteins, serum albumin and creatinine was performed by our biochemistry service, according to standard methods.

*Statistical analysis.*—Significance of the difference between volunteers and patients of Groups II and III was assessed by Student's *t* test and a *P* value of 0·05 or less was considered significant.

| | II *vs* I | III *vs* I |
|---|---|---|
| 10 min | * | * |
| 30 min | NS | * |
| 60 min | NS | NS |
| 120 min | NS | NS |

* $P < 0{\cdot}05$; NS = not significant.

FIG. 1.—Disappearance curve of *p*-aminosalicylate (PAS) from the plasma of volunteers or patients given a single 2-g oral dose of PAS. Each point represents the mean of 9 volunteers (Group I), 14 sensitive patients (Group II) and 7 resistant patients (Group III). Vertical bars represent standard errors.

Group I: $m = -0 \cdot 004690$; $b = 1 \cdot 755$; $r = -0 \cdot 9996$
Group II: $m = -0 \cdot 003952$; $b = 1 \cdot 750$; $r = -0 \cdot 9965$
Group III: $m = -0 \cdot 004024$; $b = 1 \cdot 495$; $r = -0 \cdot 9851$

FIG. 2.—Plasma half-life ($T_{1/2}$) of PAS in volunteers (Group I), sensitive patients (Group II) and resistant patients (Group III). $T_{1/2}$ was calculated from the regression line obtained from the logarithm of PAS plasma concentration versus time. The correlation coefficients ($r$) are significant.

### RESULTS

The plasma disappearance curves of unchanged para-aminosalicylate (PAS) after a single oral dose of PAS are presented in Fig. 1. In the volunteer and patient groups, the peak plasma concentration of PAS is reached 30 min after drug administration. Judging by the low PAS plasma concentration observed at 10 min, the gastrointestinal absorption of PAS seems to be delayed in all cancer patients, sensitive or resistant to chemotherapy. But the PAS concentration continues lower in resistant patients (Group III) when there is no significant difference between sensitive patients (Group II) and volunteers (Group I).

From the preceding data, regression lines were calculated, and plotted as shown in Fig. 2. Plasma half-life ($T_{1/2}$) of PAS was then estimated for the 3 groups. $T_{1/2}$ does not differ between sensitive and resistant patients, as also demonstrated by the values (m) of the slope. But compared with volunteers, $T_{1/2}$ of PAS in the patients is slightly prolonged.

Reverse curves were obtained with PAS conjugation (Fig. 3). Resistant patients (Group III) acetylated PAS to a much greater extent than volunteers or sensitive patients. In Group III, we observed an increase in APAS, up to 75% of total plasma PAS, while volunteers and sensitive patients did not acetylate more than 50% of PAS.

TABLE I.—*PAS and APAS in Total Urine of Volunteers (Group I), Sensitive Patients (Group II) and Resistant Patients (Group III), at* 120 *min Following a Single* 2-*g Oral Dose of PAS*

| | PAS (mg) Mean ± s.e. | APAS (%) Mean ± s.e. |
|---|---|---|
| Group I | 331·3 ± 26·3 | 56·4 ± 4·2 |
| Group II | 291·7 ± 38·5 | 54·9 ± 3·2 |
| Group III | 97·6 ± 12·7 | 71·4 ± 3·3 |
| II *vs* I | NS* | NS* |
| III *vs* I | $P < 0 \cdot 05$ | $P < 0 \cdot 05$ |

* NS = not significant.

TABLE II.—*Abnormal Liver Functions and Creatinine*

| | Functions | Number (and initials) of patients having abnormal values |
|---|---|---|
| * Group II | LDH | 2 (J.F.) (L-P.F.) |
| | AP | 1 (L-P.F.) |
| | SGOT | 1 (J.F.) |
| | SGPT | 1 (J.F.) |
| | Total serum proteins | 5 (A.C.) (F.L.) (M.M.) (A.G.) ( A.D.) |
| † Group III | LDH | 2 (D.L.) (L.G.) |
| | SGOT | 1 (L.G.) |
| | SGPT | 1 (L.G.) |
| | Total serum proteins | 2 (D.L.) (C.T.) |
| | Serum albumin | 3 (D.L.) (L.G.) (C.T.) |
| | Creatinine | 2 (D.L.) (C.T.) |

* Tests in 14 sensitive patients.
† Tests in 4 resistant patients.

| | II *vs* I | III *vs* I |
|---|---|---|
| 10 min | NS | NS |
| 30 min | NS | * |
| 60 min | NS | NS |
| 120 min | NS | * |

* $P < 0 \cdot 05$; NS = not significant.

FIG. 3.—Percentage of N-acetyl-*p*-aminosalicylate (APAS) recovered in plasma, from 10 to 120 min following a single 2-g oral dose of PAS. Each point represents the mean of 9 volunteers (Group I), 14 sensitive patients (Group II) and 7 resistant patients (Group III). Vertical bars represent standard errors.

In urine collected at 120 min after drug administration, there is no significant difference for PAS and APAS between Groups I and II (Table I). But, as in plasma, there is less PAS and more APAS in Group III than in volunteers.

Finally, standard tests for liver function and creatinine were done in volunteers and patients (Table II). All volunteers had normal values. Seven out of 14 in Group II had moderately abnormal functions (often evidenced in only one test), while out of 4 Group III patients tested, 3 or 4 abnormal functions were observed in 3 patients.

### DISCUSSION

The present investigation was undertaken to study the role of certain host factors such as drug distribution and metabolism in clinical resistance to cancer chemotherapy. As suggested by Connors (1974) and Dedrick *et al.* (1975), host effects might explain the ineffectiveness of certain drugs in the treatment of cancer: diminished absorption from site of administration, poor transport to the tumour, decreased biotransformation by the liver, leading to a diminution in active cytotoxic metabolites of certain drugs like cyclophosphamide, cytarabine and mercaptopurine (Chabner *et al.*, 1975).

We decided to measure *p*-aminosalicylate (PAS) metabolism for two reasons. First, the presence of cancer modifies the pharmacokinetics of many drugs, antineoplastic (Kato *et al.*, 1968*b*; Bartosek *et al.*, 1973; Benjamin, 1974; Bartosek *et al.*, 1975; Lavigne *et al.*, 1975; Donelli *et al.*, 1976) as well as other (Kato, Takanaka and Oshima, 1968*a*; Rosso, Dolfini and Donelli, 1968; Franchi and Rosso, 1969; Rosso *et al.*, 1971; Basu, Parke and

Williams, 1974*a*; Sharma and Garb, 1974; Beck, Mandel and Fabro, 1975; Nadeau and Marchand, 1975; Marchand and Nadeau, 1976). Second, some metabolic properties of PAS (Way *et al.*, 1948; Wan *et al.*, 1974) such as short plasma half-life, predominantly renal excretion and the absence of side-effects at the dose used (Weinstein, 1975) make it an interesting model drug.

In healthy volunteers (Group I) the plasma peak concentration of sodium PAS (Fig. 1) and the plasma half-life (Fig. 2) are comparable with reported values (Way *et al.*, 1948; Lavigne and Marchand, 1973; Wan *et al.*, 1974; Weinstein, 1975). The PAS concentrations (Fig. 1) in plasma of patients (Groups II and III) are lower at 10 min than those of volunteers, and seem to indicate a delay in gastrointestinal absorption of PAS. Similar observations were made with sulphacetamide in rats bearing solid tumour (Nadeau and Marchand, 1975) and in L1210 leukaemic mice (Marchand and Nadeau, 1976). Many factors could be responsible for impaired drug absorption, such as decreased mucosal blood flow to the intestine and slowing in gastric emptying (Levine, 1970), necrosis and leukaemic infiltration of the gastrointestinal tract (Matis, 1974), and occlusion of the small intestine (Gardais, François and Ronceray, 1976).

The slightly prolonged plasma half-life of PAS in patients suffering from malignant blood disease is not surprising (Fig. 2). The disappearance rate of carisoprodol (Kato *et al.*, 1968*a*), pentobarbital (Rosso *et al.*, 1971; Beck *et al.*, 1975), adriamycin (Benjamin, 1974), sulphacetamide (Nadeau and Marchand, 1975) and cyclophosphamide (Donelli *et al.*, 1976) from the plasma of cancer animals or patients was slower than that of controls. For these drugs, prolonged plasma half-life was usually explained by decreased drug-metabolizing activity in liver enzymes.

In the present experiments, we observed a significant increase of PAS acetylation (Fig. 3) in Group III patients. N-acetyl-*p*-aminosalicylic acid (APAS) is the principal metabolite of PAS (Way *et al.*, 1948; Wan *et al.*, 1974) and this conjugation of PAS with acetyl radical leads to an inactive product, as happens in most Phase II drug reactions (Bousquet, 1970). The high percentage of APAS might therefore be correlated with the low plasma level of unchanged active PAS in the plasma of Group III (Fig. 1). We may argue that the enhanced catabolism of PAS, although unexplained, is as noxious to our Group III patients as the known decreased hepatic activation of some antineoplastic drugs is to tumour-bearing animals (Kato *et al.*, 1968*b*; Bartosek *et al.*, 1975; Donelli *et al.*, 1976). It is known that antineoplastic agents are activated into cytotoxic metabolites or inactivated into degradation products mainly by the Phase I drug reactions (oxidation, reduction, hydrolysis). However, adrenocortical steroids are conjugated with sulphate or with glucuronic acid, and 6-mercaptopurine undergoes a methylation (conjugation) to give 6-MMP (Calabresi and Parks, 1975; Chabner *et al.*, 1975).

In urine collected at 120 min following drug administration (Table I), the percentage of PAS conjugated to APAS in Group I as well as the percentage (about 20) of the dose of excreted PAS are similar to those reported by Way *et al.* (1948), for healthy volunteers. Impaired excretion of PAS in Group III is possible, but is difficult to assess, because the 5% of the dose excreted after 120 min may be explained by the low plasma concentrations of PAS (Fig. 1) compared with volunteers. The slightly prolonged plasma half-life of PAS cannot explain a slower renal clearance, because Group II, who also have a slightly prolonged half-life, excrete PAS as well as volunteers do.

We must stress that our subjects were volunteers or patients of either sex, young and old. As far as the curves of plasma disappearance of PAS, acetylation and renal excretion are concerned, age and sex did not seem to have any effect, contrary to what is reported in the literature for many drugs (Bousquet, 1970; Triggs and Nation, 1975). But, in agreement with the study of Kampmann, Sinding and Jorgensen (1975), we did not find any effect of age on liver functions. In Group II (Table II), only 2 sensitive patients had 2 or 3 moderately abnormal liver functions, and 5 of these patients have only slightly low total serum proteins (5·3–5·7%). In this group, one patient suffering from lymphosarcoma had an elevated level of alkaline phosphatase, an enzyme whose elevation correlates well with this disease (Belliveau, Wiernik and Abt, 1974). Finally, the higher frequency of abnormal liver function in Group III must be pointed out. The far-advanced malignant disease is probably more responsible than the chemotherapy for the impaired liver functions. Such findings were reported earlier in patients with advanced non-hepatic cancer (Basu, Raven and Williams, 1974*b*) and it is also known that hepatic failure is a cause of death in leukaemia (Chang *et al.*, 1976). The fact that 3 out of 4 resistant patients have low serum albumin is interesting because, as mentioned by Wilkinson and Schenker (1975), depressed serum albumin level and/or prolonged prothrombin time have provided the only significant correlations between biochemical assessment of liver function and drug disposition. Hypoalbuminaemia may account for a possible diminution of albumin binding of drugs, and the smaller the extent of albumin binding, the more drug will be available for hepatic biotransformation (Koch-Weser and Sellers, 1976). The significant increase in PAS acetylation (Group III) might in part be so explained. It is also important to note that 2/4 resistant patients had elevated creatinine levels. But even if renal complications may occur in leukaemia and lymphomas (Frei *et al.*, 1963), a definite correlation with PAS excretion cannot be made.

To summarize our results, malignant blood diseases did not seem greatly to influence PAS metabolism in sensitive patients. However, in cancer patients resistant to chemotherapy, we think that changes in PAS absorption, fate and excretion might reflect, by analogy, changes in antineoplastic drug metabolism. Of course, in these patients it may be hard to dissociate resistance to chemotherapy from the advanced stage of the disease. As mentioned earlier, these patients died shortly (30–90 days) after our study and moreover, as demonstrated in tumour-bearing animals (Kato *et al.*, 1968*a*, *b*; Rosso *et al.*, 1968; Franchi and Rosso, 1969; Rosso *et al.*, 1971; Basu *et al.*, 1974*a*; Beck *et al.*, 1975; Lavigne *et al.*, 1975) drug metabolism and action were progressively modified as a function of tumour growth.

This investigation was supported by the Medical Research Council of Canada (Grant No. MA-5561).

### REFERENCES

BARTOSEK, I., MARC, V., GUAITANI, A. & GARATTINI, S. (1973) Accelerated Catabolism of Hydrocortisone in Isolated Perfused Liver of Tumorbearing Rats. *Biochem. Pharmac.*, **22**, 2429.

BARTOSEK, I., DONELLI, M. G., GUAITANI, A., COLOMBO, T., ROSSO, R. & GARATTINI, S. (1975) Differences of Cyclophosphamide and 6-Mercaptopurine Metabolic Rates in Perfused Liver of Normal and Tumour-bearing Animals. *Biochem. Pharmac.*, **24**, 289.

BASU, T. K., PARKE, D. V. & WILLIAMS, D. C. (1974*a*) Hepatic Microsomal Drug-metabolizing Enzymes in Rats Bearing Guerin Carcinoma for Various Periods. *Cytobios*, **11**, 71.

BASU, T. K., RAVEN, R. W. & WILLIAMS, D. C. (1974*b*) Some Metabolic Functions of Liver in Patients with Advanced Non-hepatic Cancer. *Oncology*, **29**, 417.

BECK, W. T., MANDEL, H. G. & FABRO, S. (1975) Physiological Disposition of Pentobarbital in Tumor-bearing Mice. *Cancer Res.*, **35**, 1333.

BELLIVEAU, R. E., WIERNIK, P. H. & ABT, A. B. (1974) Liver Enzymes and Pathology in Hodgkin's Disease. *Cancer, N.Y.*, **34**, 300.

BENJAMIN, R. S. (1974) Pharmacokinetics of Adriamycin (NSC-123127) in Patients with Sarcomas. *Cancer Chemother. Rep.*, **58**, 271.

BOUSQUET, W. F. (1970) Role of Drug Disposition in Modifying Drug Response. In: *Current Concepts in Biopharmaceutics.* Ed. J. S. Swarbrick. Toronto: Macmillan.

BRATTON, A. C. & MARSHALL, E. K. (1939) A New Coupling Component for Sulfanilamide Determination. *J. biol. Chem.*, **128**, 537.

CALABRESI, P. & PARKS, R. E. (1975) Alkylating Agents, Antimetabolites, Hormones, and Other Antiproliferative Agents. In: *The Pharmacological Basis of Therapeutics*. Ed. L. S. Goodman and A. Gilman. New York: Macmillan.

CHABNER, B. A., MYERS, C. E., COLEMAN, C. N. & JOHNS, D. G. (1975) The Clinical Pharmacology of Antineoplastic Agents. *New Engl. J. Med.*, **292**, 1107 and 1159.

CHANG, H. Y., RODRIGUEZ, V., NARBONI, G., BODEY, G. P., LUNA, M. A. & FREIREICH, E. J. (1976) Causes of Death in Adults with Acute Leukemia. *Medicine*, **55**, 259.

CONNORS, T. A. (1974) Mechanisms of Clinical Drug Resistance to Alkylating Agents. *Biochem. Pharmac.*, **23** (Suppl. No. 2), 89.

DEDRICK, R. L., ZAHARKO, D. S., BENDER, R. A., BLEYER, W. A. & LUTZ, R. J. (1975) Pharmacokinetic Considerations on Resistance to Anticancer Drugs. *Cancer Chemother. Rep.*, **59**, 795.

DONELLI, M. G., BARTOSEK, I., GUAITANI, A., MARTINI, A., COLOMBO, T., PACCIARINI, M. A. & MODICA, R. (1976) Importance of Pharmacokinetic Studies on Cyclophosphamide (NSC-26271) in Understanding its Cytotoxic Effect. *Cancer Treatment Rep.*, **60**, 395.

FRANCHI, G. & ROSSO, R. (1969) Metabolic Fate of Zoxazolamine in Tumor-bearing Rats. *Biochem. Pharmac.*, **18**, 236.

FREI, E., III, BENTZEL, C. J., RIESELBACH, R. & BLOCK, J. B. (1963) Renal Complications of Neoplastic Disease. *J. chron. Dis.*, **16**, 757.

GARDAIS, J., FRANÇOIS, H. & RONCERAY, J. (1976) Obstruction of the Small Intestine Revealing Leukemic Deposits during Chronic Myeloid Leukemia Undergoing Acute Exacerbation. *Sem. Hôp.*, **52**, 243.

KAMPMANN, J. P., SINDING, J. & JORGENSEN, I. M. (1975) Effect of Age on Liver Function. *Geriatrics*, **30**, 91.

KATO, R. TAKANAKA, A. & OSHIMA, T. (1968*a*) Drug Metabolism in Tumour-bearing Rats. II. *In vivo* Metabolisms and Effects of Drugs in Tumor-bearing Rats. *Jap. J. Pharmac.*, **18**, 245.

KATO, R., TAKANAKA, A., TAKAHASHI, A. & ONADA, K. (1968*b*) Drug Metabolism in Tumor-bearing Rats. I. Activities of NADPH-linked Electron Transport and Drug-metabolizing Systems in Liver Microsomes of Tumor-bearing Rats. *Jap. J. Pharmac.*, **18**, 224.

KOCH-WESER, J. & SELLERS, E. M. (1976) Binding of Drugs to Serum Albumin. *New Engl. J. Med.*, **294**, 311 and 526.

LANE, M. (1974) Clinical Resistance to Cancer Chemotherapy. *Biochem. Pharmac.*, **23** (Suppl. No. 2), 453.

LAVIGNE, J.-G. & MARCHAND, C. (1973) Inhibition of the Gastrointestinal Absorption of *p*-aminosalicylate (PAS) in Rats and Humans by Diphenhydramine. *Clin. pharmac. Ther.*, **14**, 404.

LAVIGNE, J.-G., BARRY, A., D'AUTEUIL, C. & DELAGE, J.-M. (1975) Increase in the Gastrointestinal Absorption and in Tissue Storage of Cyclophosphamide in L-1210 Leukaemic Mice at an Advanced Stage of the Disease. *Br. J. Cancer*, **32**, 720.

LAVIGNE, J.-G. (1976) Antineoplastic Drugs: Resistance and Metabolism. *Union Med. Canada*, **105**, 186.

LEVINE, R. R. (1970) Factors Affecting Gastrointestinal Absorption of Drugs. *Am. J. dig. Dis.*, **15**, 171.

MARCHAND, C. & NADEAU, D. (1976) The Distribution and Kinetics of Sulphacetamide in Leukaemic Mice. *Br. J. Pharmac.*, **56**, 423.

MATIS, J. D. (1974) Leukemic Changes of Gastrointestinal Tract. *N.Y. State J. Med.*, **74**, 1035.

NADEAU, D. & MARCHAND, C. (1975) Change in the Kinetics of Sulphacetamide Tissue Distribution in Walker Tumor-bearing Rats. *Drug Metab. Disp.*, **3**, 565.

ROSSO, R., DOLFINI, E. & DONELLI, M. G. (1968) Prolonged Effect of Pentobarbital in Tumor Bearing Rats. *Eur. J. Cancer*, **4**, 133.

ROSSO, R., DONELLI, M. G., FRANCHI, G. & GARATTINI, S. (1971) Impairment of Drug Metabolism in Tumor-bearing Animals. *Eur. J. Cancer*, **7**, 565.

SHARMA, G. C. & GARB, S. (1974) Influence of Cancer on the Levels of Pentobarbital in the Blood and Brain in Mice. *Gann*, **65**, 467.

TRIGGS, E. J. & NATION, R. L. (1975) Pharmacokinetics in the Aged: A Review. *J. Pharmacokin. Biopharm.*, **3**, 387.

WAN, S. H., PENTIKAINEN, P. & AZARNOFF, D. L. (1974) Bioavailability Studies on Para-aminosalicylic Acid and its Various Salts in Man. I. Absorption from Solution and Suspension. *J. Pharmacokin. Biopharm.*, **2**, 1.

WAY, E. L., SMITH, P. K., HOWIE, D. L., WEISS, R. & SWANSON, R. (1948) The Absorption, Distribution, Excretion and Fate of Para-aminosalicylic acid. *J. Pharm. exp. Ther.*, **93**, 368.

WEINSTEIN, L. (1975) Antimicrobial Agents: Drugs Used in the Chemotherapy of Tuberculosis and Leprosy. In: *The Pharmacological Basis of Therapeutics*. Ed. L. S. Goodman and A. Gilman. New York: Macmillan.

WILKINSON, G. R. & SCHENKER, S. (1975) Drug Disposition and Liver Disease. *Drug Metab. Rev.*, **4**, 139.